# Short-term respiratory outcomes in late preterm infants

**DOI:** 10.1186/1824-7288-40-52

**Published:** 2014-06-03

**Authors:** Miria Natile, Maria Luisa Ventura, Marco Colombo, Davide Bernasconi, Anna Locatelli, Cristina Plevani, Maria Grazia Valsecchi, Paolo Tagliabue

**Affiliations:** 1 Neonatology and Neonatal Intensive Care Unit, MBBM Foundation, via Pergolesi 33, 20900 Monza, Italy; 2 Department of Clinical Medicine and Prevention, Center of Biostatistics for Clinical Epidemiology, via Pergolesi 33, 20900 Monza, Italy; 3 Department of Obstetrics and Gynecology, University of Milano-Bicocca, via Pergolesi 33, 20900 Monza, Italy

**Keywords:** Late preterm infants, Respiratory outcome

## Abstract

**Objective:**

To evaluate short-term respiratory outcomes in late preterm infants (LPI) compared with those of term infants (TI).

**Methods:**

A retrospective study conducted in a single third level Italian centre (2005–2009) to analyse the incidence and risk factors of composite respiratory morbidity (CRM), the need for adjunctive therapies (surfactant therapy, inhaled nitric oxide, pleural drainage), the highest level of respiratory support (mechanical ventilation – MV, nasal continuous positive airway pressure – N-CPAP, nasal oxygen) and the duration of pressure support (hours in N-CPAP and/or MV).

**Results:**

During the study period 14,515 infants were delivered. There were 856 (5.9%) LPI and 12,948 (89.2%) TI. CRM affected 105 LPI (12.4%), and 121 TI (0.9%), with an overall rate of 1.6%. Eighty-four LPI (9.8%) and 73 TI (0.56%) received respiratory support, of which 13 LPI (1.5%) and 16 TI (0.12%) were ventilated. The adjusted OR for developing CRM significantly increased from 3.3 (95% CI 2.0-5.5) at 37 weeks to 40.8 (95% CI 19.7-84.9%) at 34 weeks. The adjusted OR for the need of MV significantly increased from 3.4 (95% CI 1.2-10) at 37 weeks to 34.4 (95% CI 6.7-180.6%) at 34 weeks. Median duration of pressure support was significantly higher at 37 weeks (66.6 h vs 40.5 h). Twin pregnancies were related to a higher risk of CRM (OR 4.3, 95% CI 2.6-7.3), but not independent of gestational age (GA). Cesarean section (CS) was associated with higher risk of CRM independently of GA, but the OR was lower in CS with labour (2.2, 95% CI 1.4-3.4 vs 3.0, 95% CI 2.1-4.2).

**Conclusions:**

In this single third level care study late preterm births, pulmonary diseases and supportive respiratory interventions were lower than previously documented. LPI are at a higher risk of developing pulmonary disease than TI. Infants born from elective cesarean sections, late preterm twins in particular and 37 weekers too might benefit from preventive intervention.

## Background

Late preterm infants (LPI), previously known as near term, are premature newborns delivered between 34 0/7 and 36 6/7 weeks of gestational age according to the definition developed by the National Institute of Child Health and Human Development Workshop [1] in 2005.

In the United States (U.S.) [2] the delivery rate of preterm births increased from 10.6% to 12.8% in 1990 to 2006, whereas the rate of LPI rose from 7.3% to 9.1% in the same period, therefore the increase of preterm births was primarily due to an increase of late preterm deliveries [3]. The decline in LPI from 9.1% in 2007 to 8.1% in 2012 and the decline in preterms from 12.8% to 11.5% respectively, reflected the effectiveness of the prevention of late preterm delivery [4] in the last six years in the U.S.

Late preterm birth increases the risk of neonatal mortality and morbidity such as hypoglycemia [5], feeding problems [6], jaundice [7], hypothermia, sepsis, seizures, compared with term birth [8,9].

Previous studies consistently revealed that LPI experience respiratory distress syndrome, transient tachypnoea of the newborn, pneumonia and persistent pulmonary hypertension, at higher rates than term infants (TI) [10,11]. This increased respiratory morbidity is related to functional immaturity of the lung structure, which can lead to impaired gas exchange and requires respiratory support [12].

The purpose of this study is to evaluate short-term respiratory outcomes in LPI compared with those in TI and to identify the risk factors of these outcomes.

## Materials and methods

This observational study was carried out in a single third level neonatal centre (S. Gerardo Hospital of Monza) from January 2005 to December 2009. The cohort of all late preterm deliveries was compared with the cohort of all full-term deliveries (37 0/7 to 41 6/7 weeks of gestation) in the same period.

The cohorts were identified on our electronic medical records (MetaVision® - iMDsoft) for all singleton and multiple live births. An informative privacy consent form was signed by each of the parents of all the patients upon admission.

Gestational age was estimated by the association of ultrasound examination before 20 weeks of gestation and the last menstrual period. Extracted data included gender, birth weight, multiple births, presence of major congenital anomalies, mode of delivery, Apgar score at minute 5, need for resuscitation, admission to the neonatal intensive care unit (NICU) and days of hospitalization.

Infants requiring respiratory care or cardio-respiratory monitoring, infants with a birth weight < 1800 g or a gestational age < 34 weeks and infants who require immediate operative procedures were admitted to the NICU. Following a clinical evaluation, the stable infants remained with their mothers in the postpartum ward.

Newborns were defined small for gestational age (SGA) if the birth weight was less than the 10th percentile [13]. Need for resuscitation was defined as need for intermittent positive pressure ventilation and/or cardiac compression and/or drug administration in the neonatal stabilization period.

Maternal characteristics included maternal age, ethnicity, need for assisted reproductive technologies (ART), maternal diseases (gestational and non-gestational diabetes mellitus, pre-eclampsia, hemolysis, elevated liver enzymes and low platelet count syndrome - HELLP syndrome, hypertension, pregnancy induced hypertension, thrombocytopenia, anaemia, hypo/hyperthyroidism, infections, connective tissue disease, gestational cholestasis, praevia or accrete placenta, poly/oligohydramnios) and the number of ultrasound and medical examinations during pregnancy. Neither tocolytic therapy nor corticosteroids for fetal lung maturation were administered at gestational age 34 0/7 weeks or above. Deliveries were categorized as vaginal, Cesarean Section (CS) during labour and CS before labour. The diagnoses of respiratory disorders were made in accordance with the following definitions:

– Respiratory distress syndrome (RDS): oxygen-dependence increasing during the first 24 h, typical radiological findings: reduced air content, reticulogranular pattern of the lungs, bronchogram.

– Transient tachypnea of the newborn (TTN): oxygen supplement requirement during the first 6 h which decreases during the subsequent 18 h, improvement in clinical condition within 6 h and chest x-rays which are either normal or show reduced translucency, infiltrates and hyperinsufflation of the lungs.

– Apnea: breathing pauses that last for >20 seconds or for >10 seconds if associated with bradycardia or oxygen desaturation.

– Persistent pulmonary hypertension (PPH): persistent elevation in pulmonary artery pressure with right-left shunt and hypoxia.

– Air leak: presence of pneumothorax and/or pneumomediastinum.

– Meconium aspiration syndrome (MAS): respiratory distress in a meconium-stained newborn and chest x-ray indicating aspiration.

– Respiratory failure: other respiratory clinical conditions not included in previous categories which needed respiratory support.

The total number of respiratory cases was included in the composite respiratory morbidity (CRM): each case may be affected by one or more of these respiratory diseases.

Data on respiratory support such as oxygen therapy administered through nasal-cannulae (nasal oxygen), nasal continuous positive airways pressure (N-CPAP), mechanical ventilation, (MV) were collected. In accordance with departmental protocol, respiratory support was provided starting with nasal oxygen or N-CPAP at the first signs of respiratory impairment. MV was limited to those infants who were unresponsive to N-CPAP or to those in need of resuscitation; they were extubated as soon as possible and N-CPAP applied. When analyzing the data, for each infant only the highest level of respiratory support was considered. The need for specific adjunctive therapies (pleural drainage, surfactant administration, inhaled nitric oxide) and the duration of pressure support (sum of hours on MV and N-CPAP) were also recorded.

Clinical characteristics of the two groups were described by mean values and standard deviations (median and interquartile ranges were used for non-normally distributed variables) or rate and percentages, according to the type of variable. The Student’s *t*-test was used to compare continuous variables between the groups (Mann–Whitney U test was used for non-normally distributed variables) and the X^2^ test and Fisher Exact test were used for categorical variables. A multivariate logistic regression model was applied to examine respiratory morbidities and need for respiratory support across gestational age controlling for mode of delivery, birth weight, gender, presence of anomalies, twin births and maternal medical disorders. Multivariate logistic regression analyses were used to assess the impact of gestational age and other possible risk factors on adverse respiratory outcomes. Significant results are those related to p values < .05 or 95% confidence interval (CI) for the odds ratio (OR) not inclusive of the unit.

## Results

During the study period 14,515 infants were delivered; 639 (4.4%) very preterm infants (<34 weeks of gestational age) and 72 (0.49%) post-term infants (>41 weeks of gestational age) were excluded from the analysis. The TI were 12,948 (89.2%) and LPI were 856 (5.9%).Twenty-eight newborns, 6 LPI and 22 TI, were also excluded from analysis as no clinical data were available due to transfer to other centres (25 newborns) or due to death at delivery (3 newborns).

The majority of LPI were born at 36 weeks (53.9%), followed by those born at 35 weeks (28.4%) and at 34 weeks (17.8%), respectively.

Maternal demographic data are shown in Table 1. A greater number of women delivering LPI were treated with assisted reproductive technologies (ART, 1.6% vs 0.5%), more women in this group underwent ultrasound procedures (5.1 ± 4 vs 3.7 ± 2) and there was a higher rate of medical disorders (37.0% vs 11.9%) during pregnancy.

**Table 1:** Maternal data by gestational age in weeks

	**34**	**35**	**36**	**LPI**	**37**	**38**	**39**	**40**	**41**	**TI**	**34-41**
	n = 151	n = 241	n = 458	**n = 850**	n = 1149	n = 2444	n = 3343	n = 3729	n = 2261	**n = 12926**	**n = 13776**
**Age**											
Mean (SD)	32.4(5.4)	31.7(6.6)	32.9(4.8)	**32.5(5.5)**	32.7(5.2)	32.5(5.1)	32.1(5.0)	32.1(5.1)	31.9(5.0)	**32.2(5.1)**	**32.2(5.2)**
Number of US^***^											
Mean (SD)	4.7(2.3)	5.0(2.8)	5.2(4.7)	**5.1(4.0)**	4.5(3.4)	4.0(2.2)	3.6(1.6)	3.5(1.5)	3.5(2.0)	**3.7(2.0)**	**3.9(2.1)**
**Ethnicity**											
Caucasian	119(89.5)	181(85.0)	357(86.9)	**657(86.8)**	933(88.0)	1953(85.8)	2722(88.1)	3097(88.6)	1852(88.0)	**10557(87.8)**	**11214(81.4)**
African	7(5.3)	10(4.7)	21(5.1)	**38(5.0)**	51(4.8)	115(5.1)	135(4.4)	155(4.4)	115(5.5)	**571(4.8)**	**609(4.4)**
Hispanic	3(2.3)	18(8.4)	22(5.4)	**43(5.7)**	41(3.9)	112(4.9)	138(4.5)	128(3.7)	71(3.4)	**490(4.0)**	**533(3.8)**
Asian	4(3.0)	4(1.9)	11(2.7)	**19(2.5)**	35(3.3)	97(4.3)	94(3.0)	114(3.3)	67(3.2)	**407(3.4)**	**426(3.1)**
ART^***^											
Yes	4(2.9)	3(1.3)	6(1.3)	**13(1.6)**	16(1.4)	12(0.5)	15(0.5)	14(0.4)	7(0.3)	**64(0.5)**	**73(0.5)**
Medical disorders^***^											
Yes	53(45.7)	83(40.5)	128(32.6)	**264(37.0)**	281(27.6)	422(18.8)	265(8.6)	288(8.4)	151(7.4)	**1407(11.9)**	**1671(12.1)**

Data are expressed as absolute numbers and proportions (%) of observed data in each category, unless otherwise specified. All tests refer to comparisons between late preterm infants (LPI) and term infants (TI). US ultrasounds; ART assisted reproductive technologies. ***P < .001, using χ^2^, Fisher or t test as appropriate.

Neonatal characteristics are shown in Table 2. In the LPI cohort there was a higher frequency of SGA (14.8% vs 9.3%), twins (26.0% vs 1.6%), malformations (2.4% vs 1.3%), an Apgar score of <7 at 5 minutes of life (1.3% vs 0.3%) and resuscitation at birth (10.5% vs 2.0%). There were significantly more LPI born from CS with and without labour (11.1% and 32.6%, respectively) compared with TI (7.7% and 8.1%, respectively).

**Table 2:** Neonatal characteristics by gestational age in weeks

	**34**	**35**	**36**	**LPI**	**37**	**38**	**39**	**40**	**41**	**TI**	**34–41**
	n = 151	n = 241	n = 458	**n = 850**	n = 1149	n = 2444	n = 3343	n = 3729	n = 2261	**n = 12926**	**n = 13776**
Birth weight^***^											
Mean (SD), g	2128(406)	2323(431)	2592(429)	**2433(463)**	2887(455)	3115(414)	3269(385)	3401(392)	3545(391)	**3292(442)**	**3289 (489)**
SGA^***^	17(11.3)	40(16.6)	69(15.1)	**126(14.8)**	163(14.2)	257(10.6)	298(8.9)	328(8.8)	156(6.9)	**1202(9.3)**	**1328(9.6)**
**Gender**											
Male	73(48.3)	124(51.5)	242(52.8)	**439(51.6)**	618(53.8)	1311(53.6)	1710(51.2)	1933(51.8)	1195(52.9)	**6767(52.3)**	**7206(52.3)**
Twin^***^	50(33.1)	66(27.4)	105(22.9)	**221(26.0)**	108(9.4)	90(3.7)	14(0.4)	0	0	**212(1.6)**	**233(1.7)**
Malformation^**^	6(4.0)	7(2.9)	7(1.5)	**20(2.4)**	27(2.4)	35(1.4)	35(1.1)	44(1.2)	23(1.3)	**164(1.3)**	**184(1.3)**
Delivery^***^											
Spontaneous	68(45.0)	116(48.1)	295(64.4)	**479(56.4)**	815(70.9)	1821(74.5)	2961(88.6)	3341(89.6)	1946(86.1)	**10884(84.2)**	**11363(82.5)**
CS without labor	53(35.1)	104(43.2)	120(26.2)	**277(32.6)**	246(21.4)	459(18.8)	201(6.0)	79(2.1)	58(2.6)	**1043(8.1)**	**1320(9.6)**
CS with labor	30(19.9)	21(8.7)	43(9.4)	**94(11.1)**	88(7.7)	164(6.7)	181(5.4)	309(8.3)	257(11.4)	**999(7.7)**	**1093(7.9)**
Apgar < 7 at 5 min^***^	5(3.3)	4(1.7)	2(0.4)	**11(1.3)**	6(0.5)	7(0.3)	8(0.2)	6(0.2)	6(0.3)	**33(0.3)**	**44(0.3)**
Resuscit. at birth^***^	19(12.6)	40(16.6)	30(6.6)	**89(10.5)**	61(5.3)	64(2.6)	48(1.4)	50(1.3)	38(1.7)	**261(2.0)**	**350(2.5)**
Admission to NICU^***^											
Yes	81(53.6)	92(38.2)	72(15.7)	**245(28.8)**	88(7.7)	93(3.8)	74(2.2)	83(2.2)	65(2.9)	**403(3.1)**	**648(4.7)**
Yes, for resp. disorders^a^	37(45.7)	38(41.3)	23(31.9)	**98(40.0)**	30(34.1)	26(28.0)	14(18.9)	21(25.3)	12(18.5)	**103(25.6)**	**201(2.2)**
**Length of stay**											
Median (IQR), days											
Total^***^	7.8(5–13.1)	6(4.3–10.8)	4.3(3.3–6.3)	**5.3(3.8–8.1)**	3.6(2.8–4.9)	3.1(2.6–4.1)	3(2.5–3.4)	2.9(2.5–3.4)	3(2.6–3.6)	**3(2.5–3.8)**	**3(2.6–4)**
No NICU^***^	5.1(4.3–6.2)	4.8(3.9–6.3)	4(3.2–5.4)	**4.3(3.4–5.8)**	3.4(2.7–4.4)	3.1(2.6–4)	2.9(2.5–3.4)	2.9(2.5–3.4)	2.9(2.6–3.6)	**3(2.5–3.7)**	**3(2.6–3.8)**
NICU^***^	12(8.4–17)	12(7.1–15.2)	10.4(7.6–16)	**11.6 (8–15.8)**	8.9(6.6–14.5)	8.8(6.1–14.3)	8.7(6.7–10.7)	7.9(5.6–9.9)	7.7(5.1–10.2)	**8.4(5.9–12.4)**	**9.2(6.7–14.3)**
NICU resp. disorders	12(8–18.2)	10.5(6.9–14)	8.2(5.2–13.6)	**10.2(7–15)**	8.5(5.5–19.2)	6.9(5.1–12.2)	8.4(6.9–10.7)	9.8(8.3–13.2)	7.9(5.6–11.5)	**8.6(6.1–13.2)**	**9.5(6.7–15)**
Mortality^***^	1(0.66)	3(1.2)	1(0.22)	**5(0.6)**	6(0.5)	2(0.08)	0(0.0)	1(0.03)	1(0.04)	**10(0.08)**	**15(0.1)**
**Age at death**											
Median (IQR), days	11	14(1–59)	8	**11(8–14)**	1(0–22)	25(23–27)	-	2	0	**1.5(0–23)**	**8(1–22.5)**

Data are expressed as absolute numbers and proportions (%) of observed data in each category, unless otherwise specified. All tests refer to comparisons between late preterm infants (LPI) and term infants (TI). ^a^Proportions (%) calculated on the total of neonates admitted to neonatal intensive care unit (NICU), ^b^P = 0.2 Mann–Whitney U test. ***P < .001, using χ^2^ or t test as appropriate, **P < .01.

Neonatal mortality was 0.6% in LPI and 0,08% in TI and the median age at death was 11(8–14) and 1.5 (0–23) days respectively. The 37-week subgroup had an overall mortality rate of 0.5% with a 17-fold higher risk of death compared with other TI (0.03%). Among the 6 mortality cases in the 37 week group, 5 had severe anomalies and 1 an early-onset neonatal sepsis. The NICU admitted 245 (28.8%) LPI, 40% of these because of respiratory disorders.

Overall, median duration of hospitalization of LPI was significantly higher, 5.3 days (3.8-8.1) than TI, 3.0 days (2.5-3.8). However, for newborn admitted to the NICU with respiratory disorders, length of stay was not significantly different whether late or full term, 10.2 days (7.0-15.1) vs 9.0 (6.4-14.3).

As shown in Table 3, CRM affected 105 LPI (12.4%), compared with 121 (0.9%) TI. CRM decreased from 25% to 6% between the gestational ages of 34 weeks to 36 weeks, and became negligible after 38 weeks. All respiratory morbidities were consistently significantly more common in LPI than in TI, except for MAS.

**Table 3:** Respiratory Morbidity and need for respiratory support by gestational age in weeks

	**34**	**35**	**36**	**LPI**	**37**	**38**	**39**	**40**	**41**	**TI**	**34-41**
	n = 151	n = 241	n = 458	**n = 850**	n = 1149	n = 2444	n = 3343	n = 3729	n = 2261	**n = 12926**	**n = 13776**
**CRM**^a***^	38 (25.2)	38 (15.8)	29 (6.3)	**105 (12.4)**	32 (2.8)	28 (1.1)	19 (0.6)	28 (0.8)	14 (0.6)	**121 (0.9)**	**226 (1.6)**
TTN^***^	13 (8.6)	18 (7.5)	18 (3.9)	**49 (5.8)**	15 (1.3)	17 (0.7)	10 (0.3)	14 (0.4)	8 (0.4)	**64 (0.5)**	**113 (0.8)**
RDS^***^	17 (11.3)	16 (6.6)	7 (1.5)	**40 (4.7)**	9 (0.8)	3 (0.1)	2 (0.1)	2 (0.1)	1(0.0)	**17 (0.1)**	**57 (0.4)**
Apnea^***^	5 (3.3)	2 (0.8)	3 (0.7)	**10 (1.2)**	3 (0.3)	2 (0.1)	2 (0.1)	2 (0.1)	1 (0.0)	**10 (0.1)**	**20 (0.1)**
Air leak^***^	5 (3.3)	3 (1.2)	3 (0.7)	**11 (1.3)**	4 (0.3)	4 (0.2)	4 (0.1)	4 (0.1)	1 (0.0)	**17 (0.1)**	**28 (0.1)**
PPH^***^	0 (0.0)	1 (0.4)	3 (0.7)	**4 (0.5)**	1 (0.1)	0 (0.0)	1 (0.0)	2 (0.1)	0 (0.0)	**4 (0.0)**	**8 (0.2)**
MAS	0 (0.0)	0 (0.0)	0 (0.0)	**0 (0.0)**	0 (0.0)	0 (0.0)	1 (0.0)	4 (0.1)	3 (0.1)	**8 (0.1)**	**8 (0.05)**
Respiratory failure^**^	3 (2.0)	2 (0.8)	0 (0.0)	**5 (0.6)**	4 (0.3)	6 (0.2)	1 (0.0)	4 (0.1)	1 (0.0)	**16 (0.1)**	**21 (0.15)**
Respiratory support^***^	33 (21.8)	31 (12.8)	20 (4.3)	**84 (9.8%)**	23 (2)	15 (0.6)	11 (0.3)	14 (0.4)	10 (0.4)	**73 (0.56)**	**157 (1.14)**
Mechanical ventilation^*^	7 (4.6)	4 (1.65)	2 (0.43)	**13 (1.5)**	5 (0.43)	2 (0.08)	2 (0.06)	5 (0.13)	2 (0.08)	**16 (0.12)**	**29 (0.21)**
Data are expressed as absolute numbers and proportions (%) of observed data in each category. All tests refer to comparisons between late preterm infants (LPI) and term infants (TI).											

^*a*^For definition of CRM and abbreviations see text.

***P < .001, using χ^2^or Fisher test as appropriate.

**P < .01, using χ^2^or Fisher test as appropriate.

*P < .05, using χ^2^or Fisher test as appropriate.

Eighty-four LPI (9.8%) and 73 TI (0.56%) received respiratory intervention, of which 13 LPI (1.5%) and 16 TI (0.12%) were ventilated.

The highest level of respiratory support needed by neonates with CRM and adjunctive respiratory therapies can be seen in Table 4. Nasal oxygen was rare in LPI compared to that in full term infants. On the other hand, a total of 68 (64.8%) LPI received N-CPAP compared to 39 (32.2%) TI. The use of MV was similar in the two groups.

**Table 4:** Highest level of respiratory support and adjunctive therapies among neonates with composite respiratory morbidity

	**34**	**35**	**36**	**LPI**	**37**	**38**	**39**	**40**	**41**	**TI**
	n = 38	n = 38	n = 29	**n = 105**	n = 32	n = 28	n = 19	n = 28	n = 14	**n = 121**
Nasal oxygen^**^	0 (0.0)	1 (2.6)	2 (6.9)	**3 (2.9)**	5 (15.6)	2 (7.1)	3 (15.8)	5 (17.9)	3 (21.4)	**18 (14.9)**
N-CPAP^***^	26 (68.4)	26 (68.4)	16 (55.2)	**68 (64.8)**	13 (40.6)	11 (39.3)	6 (31.6)	4 (14.3)	5 (35.7)	**39 (32.2)**
MV	7 (18.4)	4 (10.5)	2 (6.9)	**13 (12.4)**	5 (15.6)	2 (7.1)	2 (10.5)	5 (17.9)	2 (14.3)	**16 (13.2)**
Surfactant^*^	6 (15.8)	7 (18.4)	4 (13.8)	**17 (16.2)**	5 (15.6)	0 (0.0)	0 (0.0)	3 (10.7)	0 (0.0)	**8 (6.6)**
INO	0 (0.0)	2 (5.3)	2 (6.9)	**4 (3.8)**	3 (9.4)	0 (0.0)	0 (0.0)	2 (7.1)	0 (0.0)	**5 (4.1)**
Pleural drainage	6 (15.8)	1 (2.6)	1 (3.5)	**8 (7.6)**	5 (15.6)	1 (3.6)	0 (0.0)	1 (3.6)	1(7.1)	**8 (6.6)**
Data are expressed as absolute numbers and proportions (%) of observed data in each category. All tests refer to comparisons between late preterm infants (LPI) and term infants (TI).										

***P < .001, using χ^2^or Fisher test as appropriate.

**P < .01, using χ^2^or Fisher test as appropriate.

*P < .05, using χ^2^or Fisher test as appropriate.

**Figure 1:**
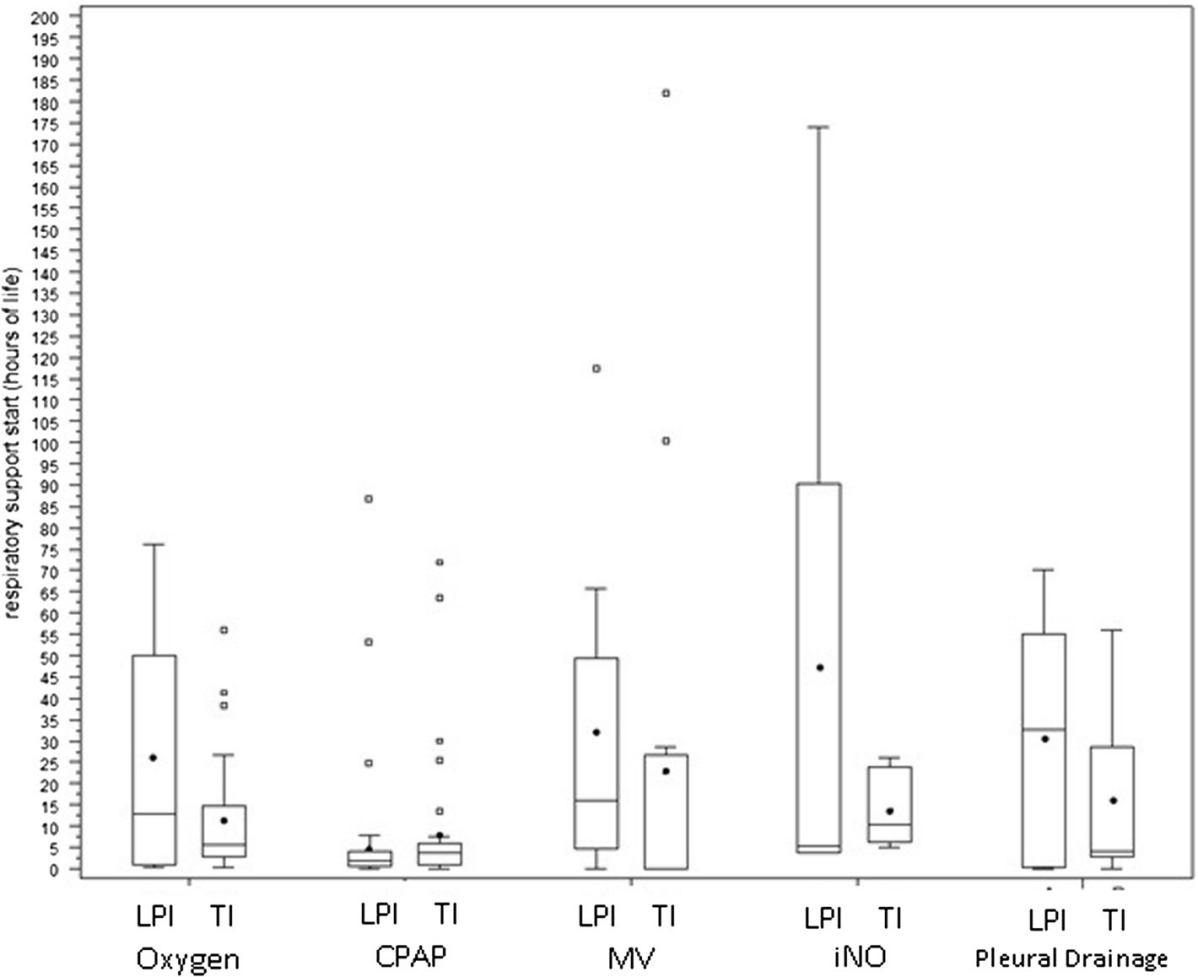
**Start of respiratory support (hours since birth) among LPI and TI with CRM.** Box plots indicate the mean value (dots) and the quartiles (horizontal segments). For abbreviations see text.

**Figure 2:**
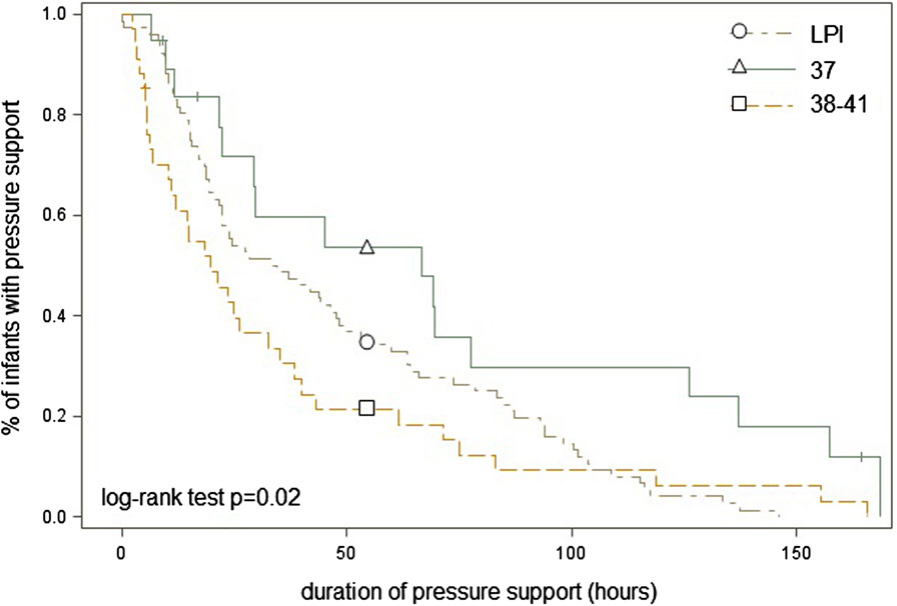
**Duration of pressure support.** Kaplan-Meier curve showing the percentage of patients on pressure support (sum of hours on MV and N-CPAP) in LPI, infants born at 37 weeks of gestation and infants born between 38 and 41 weeks of gestation.

The percentage requiring surfactant therapy in the late preterm group was significantly higher than in the term group (16.2% vs 6.6%). In the TI group, those infants born at 37 weeks gestation had a higher incidence of pleural drainage (15.6%) compared with those born at 38–41 weeks (3.4%, p 0.03), whereas other respiratory supports were not significantly different.MV (Figure 1), was started earlier in life in the TI group compared with LPI (median 0.1 vs 15.9 hours, p = 0.05).The duration of pressure support (hours in N-CPAP and/or MV) was significantly longer in 37 weeks neonates compared with that in younger (median 66.6 hrs vs 40.5 hrs) and older babies (median 66.6 hrs vs 22.4 hrs) (Figure 2).

In order to evaluate whether maternal characteristics were risk factors for late preterm birth or for CRM regardless of LPI, two logistic regression models (Table 5) were applied. For both these outcomes, maternal medical disorders and the twin pregnancies were related to a higher risk of late preterm birth (OR 3.8, 95% CI 3.1-4.7 and OR 19.3, 95% CI 14.6-25.4, respectively) and of CRM (OR 3.3, 95% CI 2.3-4.7 and OR 4.3, 95% CI 2.6-7.3, respectively).

**Table 5:** Multivariate logistic regression I

	**Odds ratio estimates (95% confidence interval)**	
	**Late preterm birth**	**CRM**
Maternal age	1.002 (0.982–1.021)	1.007 (0.971–1.043)
Maternal medical disorders no	1	1
Maternal medical disorders yes	3.8 (3.1–4.7)	3.3 (2.3–4.7)
Twins birth no	1	1
Twins birth yes	19.3 (14.6–25.4)	4.3 (2.6–7.3)

Data show occurrence of late preterm birth and of composite respiratory morbidity (CRM) by maternal characteristics (OR = 1 indicates the reference category). Data are expressed as absolute numbers and proportions (%) of observed data in each category. All tests refer to comparisons between late preterm infants (LPI) and term infants (TI).

Further analyses were carried out to assess the impact of gestational age on respiratory morbidities and respiratory support, adjusting for potential confounding factors of the mother and the infant as shown in Table 6. Compared with babies born in weeks 39–41, the OR for developing CRM gradually increases the lower the gestational age at birth: from 1.5 (not significant) at week 38, to 3.3 at week 37 and as high as 40.8 for babies born at 34 weeks. Moreover, CS without labour (OR 3.0, 95% CI 2.1-4.2) or with labour (OR 2.2, 95% CI 1.4-3.4), male gender (OR 1.4, 95% CI 1.1-1.9) and malformations (OR, 8.9 95% CI 5.3-15.1) were related to CRM independent of gestational age, while maternal medical disorders, SGA and twin pregnancies were not.

**Table 6:** Multivariate logistic regression II

	**Odds ratio estimates (95% confidence interval)**		
	**CRM**	**N-CPAP**	**MV**
Gest. age, weeks 39–40–41	1	1	1
Gest. age, week 38	1.5 (0.9– 2.4)	2.0 (0.9–4.5)	0.96 (0.3–3.1)
Gest. age, week 37	3.3 (2.0–5.5)	5.1 (2.3–11.4)	3.4 (1.2–10.0)
Gest. age, week 36	9.1 (5.1–16.2)	13.3 (5.5–32.2)	11.1 (3.2–38.8)
Gest. age, week 35	21.8 (11.5–41.5)	34.9 (13.5–90.0)	18.0 (4.2–77.5)
Gest. age, week 34	40.8 (19.7 –84.9)	55.9 (19.3–162.0)	34.4 (6.7–180.6)
Spontaneous delivery	1	1	1
CS without labor	3.0 (2.1–4.2)	3.7 (2.3–6.0)	2.9 (1.3–6.3)
CS with labor	2.2 (1.4–3.4)	2.5 (1.4–4.8)	2.1 (0.8–5.6)
Maternal medical disorders no	1	1	1
Maternal medical disorders yes	1.3 (0.9–1.8)	1.3 (0.7–1.6)	1.7 (0.8–3.4)
Twins birth no	1	1	1
Twins birth yes	0.9 (0.5–1.4)	1.0 (0.6–1.7)	0.3 (0.1–1.3)
Birth weight, g	1.0 (0.98–1.1)	1.0 (0.9–1.05)	1.1 (0.98–1.2)
Female	1	1	1
Male	1.4 (1.1–1.9)	1.5 (1.0–2.3)	1.8 (0.9–3.6)
Malformation no	1	1	1
Malformation yes	8.9 (5.3–15.1)	2.4 (0.9–6.2)	26.0 (12.5–54.1)
Small for gestational age no	1	1	1
Small for gestational age yes	1.0 (0.6–1.8)	0.6 (0.3–1.3)	2.6 (0.9–7.9)

Data compare composite respiratory morbidities (CRM), use of nasal continuous positive airway pressure (N-CPAP) and use of mechanical ventilation (MV) across gestational ages, adjusting for maternal and neonatal characteristics (OR = 1 indicates the reference category).

Taking into consideration the need for N-CPAP and the need for MV, multivariate analysis had similar results (Table 6). The adjusted ORs increased as gestational age decreased. At week 34, OR were 55.9 (95% CI 19.3-162.0) and 34.4 (95% CI 6.7-180.6) for N-CPAP and MV, respectively. At week 37, ORs were 5.1 (95% CI 2.3-11.4) and 3.4 (95% CI 1.2-10.0) for the two outcomes respectively, while at week 38 ORs were not significantly different from 1, indicating no difference in risk compared with infants born at weeks 39–41.

## Discussion

This study analyses the respiratory morbidity as well as the need for respiratory support and adjunctive therapies in a cohort of LPI and TI. In addition, risk factors for late preterm birth and respiratory outcomes were analyzed.

In contrast with birth data [1] from the United States, a lower rate of LPI (5.9% vs 9.1%) and a lower incidence of LPI among preterm infants (57% vs 75%) were observed. Although this study was carried out in a single third level centre, results are in keeping with the overall rate of preterm delivery of 6.8% in Italy [14], similar to the rate of many European countries [15] and about half that in the U.S. [16].

The recent rise in preterm births in the U.S. has been mainly due to increases in late preterm births, largely as a consequence of increases in preterm induction and preterm cesarean delivery among women at high risk for adverse pregnancy outcomes [17]. However, according to the literature, iatrogenic births in this category vary between 8% and 46% [18,19] and a recent article reports an overall 57% of “unnecessary”, avoidable late preterm births [20].

Although a rise was recorded in late preterm births between 1995 and 2005 in our centre too, this was only from 4.9% to 5.3%.

During the first 4 years of the study period, an increase of late preterm births from 5.3% to 6.6% was detected, while this returned to 5.3% in the last year. However the reasons for these fluctuations are not documented as our database did not include enough information regarding induction of labour and indications for delivery, it is not possible to distinguish between indicated and non-indicated deliveries. The figures in this study could reflect the ability of obstetric management to maintain a low level of late preterm births.

In line with the literature [21,22] this study found that women delivering LPI had significantly more medical complications than women delivering TI; the LPI were more frequently twins, SGA, malformed and born by CS.

Multivariate analysis confirmed that maternal complications and twin pregnancies were associated with late preterm birth and CRM, but not independent of gestational age. The higher risk of respiratory disorders in late preterm twins, primarily related to their preterm birth, therefore could be reduced by a careful continuation of gestation. However, recent recommendations [23] emphasize the adverse outcomes in complicated twin pregnancies lasting over 37 weeks of gestation, especially if monochorionic.

Elective CS is considered a major contributor to respiratory morbidities in both late preterm and early term neonates [24,25]. De Luca *et al*. [26] demonstrated that compared with planned vaginal delivery, infants delivered by elective CS had significantly higher mortality and respiratory morbidity. Roth-Kleiner and coworkers showed that, in term and near-term infants who developed respiratory distress following elective CS, the need for mechanical ventilation was dramatically higher than in those born after CS in labour [27].

The rate of CS without labour in the LPI group was higher than that reported by the U.S. Consortium on Safe Labour [28] (32.6% vs 23.6%), but CS in labour was lower in this study (11.1% vs 15%) and the overall rate was lower than that reported in a tertiary centre dataset by Holland *et al*. [19] (44% vs 61%). Moreover the location of the present study has one of lowest rates of total CS in Italy (overall 19% vs 38%.). Multivariate analysis also showed that mode of delivery was independently related to neonatal CRM and a protective effect of labour independent of the final mode of delivery was confirmed.

Several studies have demonstrated that LPI are at high risk of death during the neonatal period, particularly in the first few days of life [29]. The neonatal mortality rate in LPI was 7.4-fold that in TI, comparable to the gestation-specific neonatal mortality rates from other data sources [30,31]. Mortality in the 37-week group was higher than in the 38–41 groups, but the main cause was congenital malformation, as also demonstrated by Young *et al*. [32].

CRM was nearly 14-fold higher in the LPI group than in the term group. It occurred in 12.4% of the late preterm birth cohort, one of the lowest rates among those registered in other series. In the literature, there is great variability of the rate of CRM due to the heterogeneity of studies, selection criteria and definition of the outcome. CRM is often used to define frequency in pulmonary complications but it has a wide range of severity. Despite the rigorous definitions used to describe RDS and TTN, the diagnosis may still be subjective and the distinction between TTN and RDS can sometimes be unclear. Among the LPI, TTN was the most frequent disease (5.8%) followed by RDS (4.7%). This differs from other studies [33] in which RDS was the most frequent disease with a rate as high as 28.9%. The findings of the present study are more in line with those of Melamed *et al*. [34] who reported a 4.2% rate of RDS in an Israeli case–control study of LPI. Moreover a recent systematic review [30], including 22 different studies on LPI morbidity, recorded RDS at 5.3%. However, the emerging data was that in the overall population of 34 weeks to 41 weeks gestation the incidence of CRM was 1.6%. RDS and TTN accounted for 1.4%. These rates are lower than those reported in the recent literature [28,34,35] and this could be related to the lower incidence of LPI in the centre where this study took place.

The need for respiratory support for each gestational age was analyzed. Among LPI, a lower rate of MV (1.5%) was found compared with that reported in previous studies (up to 8%) [28,35]. To what extent this was due to less severe illnesses, perhaps to better prenatal management, rather than a more gentle approach to respiratory failure, is difficult to determine. Unfortunately there is no data on the administration of antenatal corticosteroids prior to the 34th week. On the other hand, the caution concerning intubation was also confirmed in the cohort of TI in which the incidence of mechanical ventilation was 0.2%, against 1.1% of the data from the U.S. Consortium on Safe Labour or 1.2% of the data reported by Teune *et al*. in a recent review [30]. Respiratory assistance in newborns affected by CRM was also evaluated in the present study. Once respiratory distress has arisen, the risk of undergoing mechanical ventilation is no different between the LPI group and the TI. This could be read as an effect of a similar, unexpected, pathogenetic mechanism underlying the respiratory disease in both groups. This finding may suggest the introduction of preventive practices (such as antenatal corticosteroids) common to the two groups. The reduced use of nasal oxygen in the LPI group compared to that in the TI probably reflects the unit’s preference for N-CPAP instead of oxygen by nasal cannulae the lower the gestational age.

The respiratory complications seen in the study appeared mild and temporary: the median duration of respiratory pressure support for the LPI group was 40.5 hours. The median length of NICU stay for respiratory disorders was 9 days.

In agreement with recent findings, all types of neonatal respiratory morbidity as well as the need for respiratory support and adjunctive therapies decreased significantly with gestational age until 39 weeks. Each week gained until 39 weeks’ gestation reduces the risk of respiratory morbidity by half.

At 37 weeks, the estimated risk for respiratory morbidity, the need for N-CPAP and the need for MV were still greater than at 39–41 weeks. Moreover the duration of any pressure support was significantly longer in 37- week neonates compared to 34–36 and 38–41 week babies. In the population of this analysis it was noted that the number of 37-week births should be reduced by 63 in order to lower the need for respiratory support by 1. This represents a considerable undertaking for a result of questionable relevance of the reduction of a mild and temporary disease. Moreover it could expose infants to other risks related to the continuation of pregnancy.

## Conclusions

This study confirms that LPI are at a higher risk than TI of developing a pulmonary disease. However, the impact of respiratory diseases on the overall population is lower than previously reported. This is probably related to the respiratory strategy of intervention that endorses less invasive respiratory assistance, as well as to the low rate of late preterm births. Multivariate analysis identified late preterm twins, infants born from elective cesarean section and 37 weekers as the patients at high risk of respiratory impairment: this population might benefit from delayed delivery and from pharmacological lung maturation.

Nevertheless, a generic program aimed at achieving further reduction of late preterm births or cesarean sections in a population already at low risk of respiratory disorders, must seriously take into account the competing risk of other adverse events. In an endeavor to reduce non-indicated late preterm, early term births and cesarean sections, a quality-improvement initiative tailored to this specific environment is called for.

## Abbreviations

CI: Confidence interval; N-CPAP: Nasal continous positive airways pressure; CRM: Composite respiratory morbidity; CS: Cesarean section; GA: Gestational age; iNO: Inhaled nitric oxide; LPI: Late preterm infants; MAS: Meconium aspiration syndrome; MV: Mechanical ventilation; NICU: Neonatal intensive care unit; OR: Odd ratio; PPH: Persistent pulmonary hypertension; RDS: Respiratory distress syndrome; SGA: Small for gestational age; TI: Term infants; TTN: Transient tachypnea of the newborn.

## Competing interests

The authors declare that they have no financial neither non-financial competing interests

## Authors’ contributions

MN conceptualized and designed the study, drafted the initial manuscript and approved the final manuscript as submitted. MLV conceptualized and designed the study, reviewed and revised the manuscript and approved the final manuscript as submitted. MC kept the study data base, described the data, drafted the initial manuscript and approved the final manuscript as submitted. DB performed the statistical analyses, critically reviewed the manuscript and approved the final manuscript as submitted. AL critically reviewed the manuscript and approved the final manuscript as submitted. CP carried out the initial analyses and approved the final manuscript as submitted. MGV supervised the study design and the statistical analyses, drafted the initial manuscript and approved the final manuscript as submitted. PT conceptualized and designed the study, critically reviewed the manuscript and approved the final manuscript as submitted. All authors read and approved the final manuscript.

## References

[B1] Raju TN Higgins RD Stark AR Leveno KJ Optimizing care and outcome for late-preterm (near-term) infants: a summary of the workshop sponsored by the National Institute of Child Health and Human Development Pediatrics 2006 118 Suppl 3 1207 1214 16951017 10.1542/peds.2006-0018

[B2] National Center for Health Statistics Public Use Data Tapes. Natality Data Set: 1992–2002 2005 Hyattsville, MD: US Department of Health and Human Services, Centers for Disease Control and Prevention

[B3] Raju TN Epidemiology of late preterm (near-term) births Clin Perinatol 2006 33 751 763 10.1016/j.clp.2006.09.009 17148002

[B4] Hamilton BE Martin JA Ventura SJ Births: Preliminary data for 2012 Natl Vital Stat Rep 2013 62 3 24321416

[B5] Adamkin DH Committee on Fetus and Newborn. Postnatal glucose homeostasis in late-preterm and term infants Pediatrics 2011 127 3 575 579 21357346 10.1542/peds.2010-3851

[B6] Academy of Breastfeeding Medicine ABM clinical protocol #10: breastfeeding the late preterm infant (34(0/7) to 36(6/7) weeks gestation) (first revision June 2011) Breastfeed Med 2011 6 151 156 21631254 10.1089/bfm.2011.9990

[B7] Watchko JF Hyperbilirubinemia and bilirubin toxicity in the late preterm infant Clin Perinatol 2006 33 839 852 10.1016/j.clp.2006.09.002 17148008

[B8] Gouyon JB Iacobelli S Ferdynus C Bonsante F Neonatal problems of late and moderate preterm infants Semin Fetal Neonatal Med 2012 17 146 152 10.1016/j.siny.2012.01.015 22349153

[B9] Ramachandrappa A Rosenber ES Wagoner S Jain L Morbidity and mortality in late preterm infants with severe hypoxic respiratory failure on extracorporeal membrane oxygenation J Pediatr 2011 159 192 198 10.1016/j.jpeds.2011.02.015 21459387 PMC3134553

[B10] Leone A Ersfeld P Adams M Meyer PS Bucher HU Arlettaz R Neonatal morbidity in singleton late preterm infants compared with full-term infants Acta Paediatr 2012 101 6 10 10.1111/j.1651-2227.2011.02459.x 21895764

[B11] Cheng YW Kaimal AJ Bruckner TA Hallaron DR Caughey AB Perinatal morbidity associated with late preterm deliveries compared with deliveries between 37 and 40 weeks of gestation BJOG 2011 118 12 1446 1454 10.1111/j.1471-0528.2011.03045.x 21883872 PMC3403292

[B12] Raju TN Developmental physiology of late and moderate prematurity Semin Fetal Neonatal Med 2012 17 126 131 10.1016/j.siny.2012.01.010 22317884

[B13] Bertino E Spada E Occhi L Coscia A Giuliani F Gagliardi L Gilli G Bona G Fabris C De Curtis M Milani S Neonatal Anthropometric Charts: The Italian neonatal study compared with other European studies JPGN 2010 51 353 361 20601901 10.1097/MPG.0b013e3181da213e

[B14] Italian Ministry of Health CeDap, Birth analyisis in Italy 2009

[B15] MacDorman MF Mathews TJ BirthStats: percentage of preterm births, United States and selected European countries, 2004 Birth 2010 37 2 168 10.1111/j.1523-536X.2010.00397.x 20557540

[B16] Martin JA Hamilton BE Sutton PD Ventura SJ Mathews TJ Osterman MJ Births: final data for 2008 Natl Vital Stat Rep 2010 59 1 72 22145497

[B17] Joseph KS Demissie K Kramer MS Obstetric intervention, stillbirth and preterm birth Semin Perinatol 2002 26 250 259 10.1053/sper.2002.34769 12211615

[B18] Lubow JM How HY Habli M Maxwell R Sibai BM Indications for delivery and short term neonatal outcomes in late preterm as compared with term births Am J Obstet Gynecol 2009 200 30 33 10.1016/j.ajog.2008.09.022 19136092

[B19] Holland MG Refuerzo JS Ramin SM Saade GR Blackwell SC Late preterm birth: how often is it avoidable? Am J Obstet Gynecol 2009 201 404 e1-4 19716546 10.1016/j.ajog.2009.06.066

[B20] Gyamfi-Bannerman C Fuchs KM Young OM Hoffman MK Nonspontaneous late preterm birth: etiology and outcomes Am J Obstet Gynecol 2011 205 456 e1-6 22035950 10.1016/j.ajog.2011.08.007

[B21] Shapiro-Mendoza CK Tomashek KM Kotelchuck M Barfield W Nannini A Weiss J Declercq E Effect of late-preterm birth and maternal medical conditions on newborn morbidity risk Pediatrics 2008 121 2 223 232 10.1542/peds.2006-3629 18245397

[B22] Gouyon JB Vintejoux A Sagot P Burguet A Quantin C Ferdynus C Neonatal outcome associated with singleton birth at 34–41 weeks of gestation Int J Epidemiol 2010 39 3 769 776 10.1093/ije/dyq037 20304783

[B23] Medically indicated late-preterm and early-term deliveries Committee Opinion No. 560. American College of Obstetricians and Gynecologists Obstet Gynecol 2013 121 908 910 10.1097/01.AOG.0000428648.75548.00 23635709

[B24] Gerten KA Coonrod DV Bay RC Chambliss LR Cesarean delivery and respiratory distress syndrome: does labour make a difference? Am J Obstet Gynecol 2005 193 1061 1064 10.1016/j.ajog.2005.05.038 16157112

[B25] Hansen AK Wisborg K Uldbjerg N Henriksen TB Risk of respiratory morbidity in term infants delivered by elective caesarean section: cohort study BMJ 2008 336 85 87 10.1136/bmj.39405.539282.BE 18077440 PMC2190264

[B26] De Luca R Boulvain M Irion O Berner M Pfister RE Incidence of early neonatal mortality and morbidity after late-preterm and term cesarean delivery Pediatrics 2009 123 6 e1064 e1071 10.1542/peds.2008-2407 19482739

[B27] Roth-Kleiner M Wagner BP Bachmann D Pfenninger J Respiratory distress syndrome in near-term babies after caesarean section Swiss Med Wkly 2003 133 19–20 283 288 12844271 10.4414/smw.2003.10121

[B28] Hibbard JU Wilkins I Sun L Gregory K Haberman S Hoffman M Kominiarek MA Reddy U Bailit J Branch DW Burkman R Gonzalez Quintero VH Hatjis CG Landy H Ramirez M VanVeldhuisen P Troendle J Zhang J Consortium on Safe Labour Respiratory morbidity in late preterm births JAMA 2010 304 4 419 425 20664042 10.1001/jama.2010.1015 PMC4146396

[B29] Kramer MS Demissie K Yang H Platt RW Sauvé R Liston R The contribution of mild and moderate preterm birth to infant mortality, Fetal and Infant Health Study Group of the Canadian Perinatal Surveillance System JAMA 2000 284 843 849 10.1001/jama.284.7.843 10938173

[B30] Teune MJ Bakhuizen S Gyamfi Bannerman C Opmeer BC Van Kaam AH Van Wassenaer AG Morris JM Mol BW A systematic review of severe morbidity in infants born late preterm Am J Obstet Gynecol 2011 205 4 374 21864824 10.1016/j.ajog.2011.07.015

[B31] Khashu M Narayanan M Bhargava S Osiovich H Perinatal outcomes associated with preterm birth at 33 to 36 weeks’ gestation: a population-based cohort study Pediatrics 2009 123 1 109 113 10.1542/peds.2007-3743 19117868

[B32] Young PC Glasgow TS Li X Guest-Warnick G Stoddard G Mortality of late-preterm (near-term) newborns in Utah Pediatrics 2007 119 3 e659 e665 10.1542/peds.2006-2486 17332185

[B33] Wang ML Dorer DJ Fleming MP Catlin EA Clinical outcomes of near-term infants Pediatrics 2004 114 2 372 376 10.1542/peds.114.2.372 15286219

[B34] Melamed N Klinger G Tenenbaum-Gavish K Herscovici T Linder N Hod M Yogev Y Short-term neonatal outcome in low-risk, spontaneous, singleton, late preterm deliveries Obstet Gynecol 2009 114 253 260 10.1097/AOG.0b013e3181af6931 19622985

[B35] Kitsommart R Janes M Mahajan V Rahman A Seidlitz W Wilson J Paes B Outcomes of late- preterm infants: a retrospective, single-center, Canadian study Clin Pediatr (Phila) 2009 48 8 844 850 10.1177/0009922809340432 19596865

